# Loss of Myh14 Increases Susceptibility to Noise-Induced Hearing Loss in CBA/CaJ Mice

**DOI:** 10.1155/2016/6720420

**Published:** 2016-12-22

**Authors:** Xiaolong Fu, Linqing Zhang, Yecheng Jin, Xiaoyang Sun, Aizhen Zhang, Zongzhuang Wen, Yichen Zhou, Ming Xia, Jiangang Gao

**Affiliations:** ^1^School of Life Science and Key Laboratory of the Ministry of Education for Experimental Teratology, Shandong University, Jinan 250100, China; ^2^Department of Otolaryngology-Head and Neck Surgery, The Second Hospital of Shandong University, Jinan 250033, China

## Abstract

MYH14 is a member of the myosin family, which has been implicated in many motile processes such as ion-channel gating, organelle translocation, and the cytoskeleton rearrangement. Mutations in MYH14 lead to a DFNA4-type hearing impairment. Further evidence also shows that MYH14 is a candidate noise-induced hearing loss (NIHL) susceptible gene. However, the specific roles of MYH14 in auditory function and NIHL are not fully understood. In the present study, we used CRISPR/Cas9 technology to establish a Myh14 knockout mice line in CBA/CaJ background (now referred to as Myh14^−/−^ mice) and clarify the role of MYH14 in the cochlea and NIHL. We found that Myh14^−/−^ mice did not exhibit significant hearing loss until five months of age. In addition, Myh14^−/−^ mice were more vulnerable to high intensity noise compared to control mice. More significant outer hair cell loss was observed in Myh14^−/−^ mice than in wild type controls after acoustic trauma. Our findings suggest that Myh14 may play a beneficial role in the protection of the cochlea after acoustic overstimulation in CBA/CaJ mice.

## 1. Introduction

Noise-induced hearing loss (NIHL) has now become one of the most prevalent occupational injuries reported [1]. Long-term exposure to high intensity noise can cause this sensorineural hearing disorder. Currently, it has been estimated that about 500 million individuals suffer from this hazard in the world [2]. NIHL is a preventable deficit, but it is difficult to reverse it once it occurs since the lost mammalian sensory cells cannot regenerate [3–6]; therefore, understanding its pathogenesis has become a very important task for researchers. Moreover, there have been great efforts to clarify the molecular and biochemical mechanisms involved in NIHL.

Studies have shown that acoustic overstimulation could lead to the pathogenesis and biochemical changes that result in hearing loss [7, 8]. The continued and evolving research involving NIHL has determined that there is a close relationship between the occurrence of noise-induced deafness and changes in some genes, cell metabolism, cell apoptosis [9], and so forth. The pathogenesis of NIHL is very complicated, and the exact mechanism is unknown. Generally, it is the outcome of the interaction between genetic and environmental factors [9–11]. In spite of many efforts, the research progress of NIHL has been slow, and it is difficult to study NIHL in humans. No heritability studies have been performed because families exposed to identical noise conditions are almost impossible to collect [12]. Gene modified mice are good models for studying the mechanism of NIHL. In recent years, some genes have been found to affect the susceptibility to noise in animal models [13, 14]. Several of the knockout mouse lines that have been developed, including Pjvk^−/−^ [15],* PMCA2* ^+/−^ [16],* P2RX2* ^−/−^ [17], and* CDH23* ^+/−^ [18], were determined to be more sensitive to noise than their wild type controls. Meanwhile, more studies are beginning to search for new NIHL susceptibility genes, and hundreds of single nucleotide polymorphism (SNP) loci have been found in genes involved in different pathways of the inner ear. An extended analysis of 644 SNPs in 53 candidate genes was performed in two independent (Swedish and Polish) populations; and two SNPs (rs667907 and rs588035) in MYH14 resulted in a positive association in the Polish sample set and significant interaction with noise exposure level in the Swedish sample set [19, 20]. This result suggested that MYH14 is likely to be a NIHL susceptibility gene [4, 19].

MYH14, also known as nonmuscle II heavy chain (NMHCII-C), together with two other nonmuscle chains (MYH9 and MYH10), is a member of the myosin family, which have been implicated in many motile processes such as ion-channel gating, organelle translocation, and cytoskeleton rearrangement [21, 22]. Mutations in MYH14 lead to a DFNA4-type hearing impairment. Interestingly, MYH14 shares great similarities with MYH9 and MYH10 in structure [22]. MYH14 has been shown to play roles in neuritogenesis and maintenance of apical cell junctions in epithelial cells within the cochlea [23, 24]. However, the relationship between MYH14 and NIHL is still obscure, and the role they play in NIHL needs further analysis [12].

In our study, we applied the CRISPR/Cas9 technology to establish a Myh14 knockout mouse line (Myh14^−/−^) using the CBA/CaJ background strain. Then, we investigated the hearing threshold and morphological changes in these mice under normal conditions or under noise exposure. We found that Myh14^−/−^ mice did not exhibit significant hearing loss until five months of age. Moreover, Myh14^−/−^ mice were more vulnerable to high intensity noise compared to control mice. More significant outer hair cell (OHC) loss was observed in Myh14^−/−^ mice after acoustic trauma. These data indicate that the absence of Myh14 may increase susceptibility to NIHL and that Myh14 may play a beneficial role in the protection of the cochlea after acoustic stimulation in the CBA/CaJ mouse line.

## 2. Materials and Methods

### 2.1. Ethical Statement

The use of animals in this study and the experimental procedures were approved by the Animal Ethics Review Committee of Shandong University. Animal management was performed strictly in accordance with the standards of the Animal Ethics Committee of Shandong University (Permit Number: ECAESDUSM 20123004).

### 2.2. Generation of MYH14^−/−^ Mice

Myh14-deficient mice were generated using the CRISPR-Cas9 genome-editing technology and were maintained on the CBA/CaJ background. Both pX330 and pST1374 were obtained from Addgene (Plasmid ID: #42230, #44758, resp.). The CRISPR-Cas9 genome-editing technology in mice was used as previously described [25, 26]. In brief, a pair of oligonucleotides for the target sequence (5′-CCTGAAGAAAGAGCGCAATA-3′) was annealed and ligated to PX330 digested with* BbsI*. Then sgRNA was produced by in vitro transcription (T7 as promoter) using the MEGAshortscript kit (Ambion, Am1354, USA). The hCas9 mRNA was derived from pST1374-N-NLS-flag-linker-cas9, synthesized using the mMESSAGE mMACHINE T7 kit (Ambion, Am1345, USA), and polyadenylated with a polyA tailing kit (Life Technologies, USA). Both the sgRNA and the Cas9 mRNA were purified using the MEGAclear kit (Ambion, AM1908, USA) and eluted in RNase-free water.

CBA/CaJ female mice were superovulated and mated with CBA/CaJ male mice. Then, the female mice were sacrificed and the fertilized eggs were removed from the oviducts. The purified sgRNAs (50 ng/*μ*L) and* hCas9 *mRNAs (100 ng/*μ*L) were coinjected into the cytoplasm of pronuclear stage eggs. Following the injected pronuclear stage, eggs were incubated for ten minutes. Then, the eggs were transferred into the oviducts of pseudopregnant CD1 female mice. Genomic DNA was extracted from the tails of the newborn pups. The genomic DNA fragment around the gRNA target site was amplified by PCR using two sets of primers: Myh14 forward, 5′-ACCTCGTGCTTGTTCAG-3′, and Myh14 reverse 5′-TGTCTTCAGCAGGGTGT-3′. The PCR products obtained were sequenced directly or cloned using the T/A cloning method and then sequenced to identify the mutation.

### 2.3. Analysis of Potential off-Target Mutations

Off-target effects may exist in CRISPR/Cas9 technology. In response to this possibility, we used the CRISPR design tool available on the web (http://crispr.mit.edu), to search for the off-target locus. Five potential off-target sites for the Myh14 gene were found. The primers for the off-target analysis were as follows: Sin3b 5′-GCCAGGAGGTATATGAGAAC-3′ and 5′-GAAATTTCCCCAGGAATGGACTGACA-3′; Pcdh9 5′-GTGTACTTATAGCACTCACC-3′ and 5′-CTAACGCGGAAACACCTCAC-3′; Patl1 5′-CCACTGAGCCTTTCCTACCTTC-3′ and 5′-GTAAATTTAGAAATTTTATTTT-3′; Arhgap27 5′-GAAGCTAGGCCAGCGGGCGAAAG-3′ and 5′-CTGCCCATGGGCGGGGCTG-3′; and Olfr1333 5′-GCGCTATACTGTCATCCTCAAC-3′ and 5′-GCAAGCCAAGCTACGCACTG-3′. The genomic DNA fragment from the newborn pups was amplified using the PCR primers mentioned above. Then, the PCR products obtained were sequenced directly or cloned using the T/A cloning method and sequenced to identify the mutation.

### 2.4. Preparation of Protein Extracts and Western Blot Analysis

Mice were decapitated; the cochlea were quickly removed from the skull and homogenized in ice-cold cell lysis buffer (10 mM Tris, pH = 7.4, 1% Triton X-100, 150 mM NaCl, 1 mM EDTA, and 0.2 mM PMSF), then lysed for 30 min on ice, and centrifuged at 10,000 ×g at 4°C for 30 min. The supernatant was then collected, and the protein concentration was measured using a BCA kit. The samples were mixed with loading buffer and heated at 100°C for 5 min and stored at −20°C. Protein samples were separated by a 10% sodium dodecyl sulfate (SDS) polyacrylamide gel and transferred onto a PVDF membrane. The membrane was blocked in 5% nonfat dry milk in TBS-T at room temperature for 1 h and incubated with primary antibodies at 4°C overnight. After washing with TBS-T (three times for 10 min each at room temperature), membranes were incubated with an anti-rabbit HRP-conjugated secondary antibody (1 : 8000, Cell Signaling, USA) diluted in 5% nonfat dry milk in TBS-T at room temperature for 1 h. Next, membranes were washed in TBS-T (three times for 10 min each) and bands were detected using ECL Western blot detection kit (Thermo, USA). Membranes were incubated with the following primary antibodies diluted in 5% bovine serum albumin (BSA): rabbit anti-Myosin-IIa (1 : 1000, Cell Signaling, USA), rabbit anti-Myosin-IIb (D8H8) (1 : 1000, Cell Signaling, USA), rabbit anti-Myosin-IIc (D4A7) (1 : 1000, Cell Signaling, USA), and rabbit anti-*β*-actin (1 : 5000, Bioworld, China).

### 2.5. Auditory Brainstem Response (ABR) Measurement

Mice were deeply anesthetized with pentobarbital sodium (50 mg/kg body weight) by intraperitoneal injection and the body temperature was maintained at 37°C using a heat pad. Testing was performed in a sound-isolated room. Three needle electrodes were inserted subcutaneously in the anesthetized mice: one was inserted between the ears at the forehead, one was underneath the left external ear, and one was at the back near the tail. Click and tone burst stimuli at frequencies of 4, 8, 16, and 32 kHz were generated and responses were recorded using a Tucker-Davis Technologies System (TDT, USA) workstation running the SigGen32 software (TDT, USA). Auditory thresholds (dB SPL) were defined by reducing the sound intensity in5 dB steps from 90 dB to 10 dB. The ABR threshold was defined as the lowest sound intensity sufficient to elicit the first wave clearly.

### 2.6. RNA Isolation and Real-Time PCR

In parallel experiments, total RNA was extracted from mouse cochleae at a defined time point after noise exposure using TRIzol Reagent (Invitrogen) according to the provided directions. Total RNA yield and purity were assessed with Eppendorf BioPhotometer plus. All samples had A260/280 ratios of 1.9–2.1 and showed two sharp peaks corresponding to the 18S and 28S RNA on electropherograms. Then cDNA was synthesized using PrimeScript RT reagent kit with gDNA Eraser (Takara). Quantitative PCR was performed on a Bio-Rad real-time thermal cycling system with Power SYBR Green PCR Master Mix (Takara). The amplification reaction mixture (20 *μ*L) contained 800 nmol of each primer in the SYBR Green system. The quality control for the mRNA quantification was performed using three integrated control assays in the PCR array: a reverse transcription control, a positive PCR control, and a genomic DNA control. All PCR runs passed the control tests. Data were analyzed using Bio-Rad CFX manager software. The primers utilized in this study are shown as follows: Myh9 F: ACAATGGAGGCCATGAGAAT, Myh9 R: GAGATGACCCGCAGCAAG, Myh10 F: GGAGGACACCCTAGACACCA, and Myh10 R: CCACTTCCTGCTCACGTTTT

### 2.7. Noise Exposure

Mice were kept awake and placed in a stainless steel wire cage in the center of an open-field acoustic chamber. Then, they were exposed to 2–10 kHz band noise at an intensity of 105 dB SPL for 4 h to induce a temporary change in auditory threshold shifts. The sound was generated by a noise generator (SF-06, Random Noise Generator, RION, USA), amplified by a power amplifier (CDi 1000 Power Amplifier, Crown, USA), and delivered to microphones. The noise sound files were created and equalized with audio editing software (audacity portable). Sound levels were calibrated at multiple locations within the sound chamber to ensure uniformity of the stimulus.

### 2.8. Immunostaining

Immunostaining of the cochleae was performed as previously described [27]. Cochlea were fixed in 4% formaldehyde in 10 mM phosphate-buffered saline (PBS) at 4°C overnight and decalcified in 10% EDTA in 10 mM PBS at room temperature for at least one day. For sectioning, the cochleae were dehydrated with 15% sucrose for 2 h and then 30% sucrose overnight at 4°C. Samples were embedded in Tissue-Tek OCT compound and frozen in liquid nitrogen and then sectioned into 10 *μ*m thick slices. For whole-mount immunostaining, the organ of Corti's sensory epithelium was isolated from the cochleae and divided into apical, middle, and basal turn sections to then permeabilize the samples in 0.5% Triton X-100 in PBS at room temperature for 15 min. The sections or cochlea samples were washed in PBS and then blocked in 10% goat serum in PBS at 37°C for 30 min. The samples were incubated with a primary antibody at 4°C overnight. After washing with PBS, followed by further incubation with an anti-rabbit TRITC-conjugated secondary antibody diluted in PBS at 37°C for 1 h, followed by Alexa Fluor 488-conjugated phalloidin (Sigma-Aldrich, USA) at 37°C for 30 min and 4′,6-diamidino-2-phenylindole (DAPI) at 37°C for 10 min, immunofluorescence images were collected using a confocal laser-scanning microscope. Cochleae were incubated with the following primary antibodies diluted in PBS: rabbit anti-Myosin-IIa (1 : 100, Cell Signaling, USA), rabbit anti-Myosin-IIb (D8H8) (1 : 100, Cell Signaling, USA), rabbit anti-Myosin-IIc (D4A7) (1 : 50, Cell Signaling, USA), rabbit anti-ZO-1 (1 : 400, Invitrogen, USA), and rabbit anti-E-cadherin (1 : 200, Cell Signaling, USA). Goat anti-rabbit TRITC-conjugated secondary antibodies (1 : 200) were from Invitrogen, USA.

### 2.9. Histological Analysis

Cochlea samples were fixed and decalcified using a procedure similar to that used for the immunostaining assay, dehydrated by an ethanol series ranging from 30% to 100%, and embedded in paraffin to then be sectioned at a thickness of 10 *μ*m. Sections were deparaffinized by an ethanol series ranging from 100% to 30%, stained with hematoxylin and eosin (H&E), and viewed under light microscopy (Nikon YS100, Japan).

### 2.10. Quantitative Assessment of HC Loss

Mice were sacrificed 2 weeks after noise exposure and the cochlear epithelia were immunostained for HC counts. Briefly, the apical, middle, and basal parts of the basilar membrane were counted and labeled with Alexa Fluor 488-conjugated phalloidin to outline HCs and their stereocilia for quantitative assessment. The numbers of missing OHCs were counted and the ratio of missing OHCs was expressed as a percentage.

### 2.11. Statistical Analysis

All experiments were performed at least three times. Data are expressed as mean ± SD. Repeated-measures ANOVA was performed to evaluate the difference in ABR threshold shifts, one-way ANOVA or *t*-test was selected to test the MYH14 expression and cochlear hearing loss, and statistical analysis was performed using GraphPad software. For all tests, a value of *P* < 0.05 was considered to be statistically significant.

## 3. Results

### 3.1. CRISPR/Cas9-Mediated Generation of the Myh14^−/−^ Mice Line

**Figure 1:**
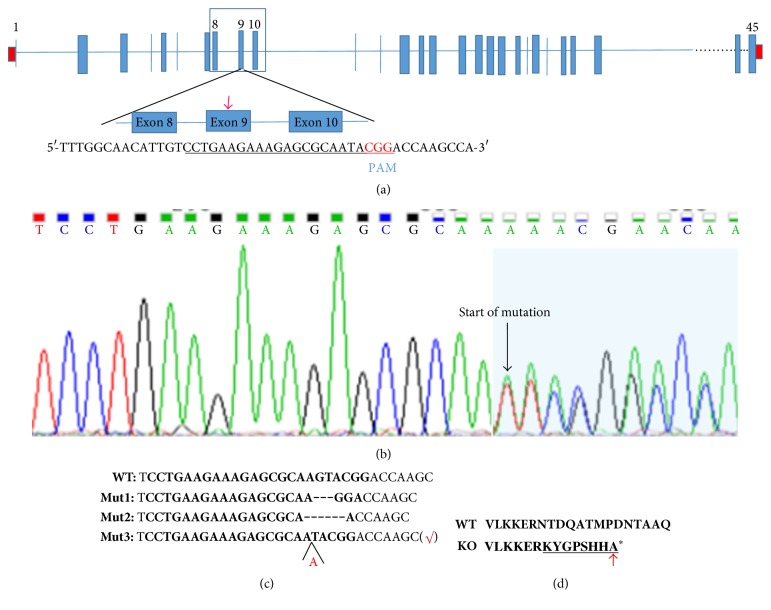
CRISPR/Cas9-mediated generation of Myh14 knockout mice. (a) Schematic diagram of sgRNA at Myh14 Exon 9 locus (indicated by the red arrow). The sgRNA sequence is underlined in black, and the PAM sequence is shown in red. (b) Sequencing chromatograms of Myh14^−/−^ mice. The sequence at the start of the mutated site becomes scrambled. (c) Three types of mutations (3, 6 bp deletions and 1 bp insertion) were produced. Type 3 frameshift mutation was chosen for further analysis. (d) Frameshift mutation of Myh14^−/−^ mice. The mutation is underlined in black. “*∗*” indicates a premature stop codon. The translation of the protein is terminated at the point of the arrow.

To investigate the functions of MYH14 in hearing, the CRISPR/Cas9 genome-editing technology was used to destroy the Myh14 gene in mice. In brief, a single guide RNA (sgRNA) containing a 20 nt target sequence and the endonuclease Cas9 are essential for successful targeting. When the sgRNA guides Cas9 to the target sequence, a double-strand break will be generated. The double-strand break produces indels (insertions and deletions), which then produce frameshift mutations. Exon 9 of the Myh14 gene is the CRISPR-amenable target, as shown in Figure 1(a). The Cas9 mRNA and the sgRNA, which were produced by in vitro transcription, were microinjected into the pronuclear stage of mouse embryos. Eighteen days after transplantation, 37 pups were born. These mice were designated as F0. Among these 37 pups, 16 pups contained the mutation, as determined by genotyping sequence analysis, and surprisingly, all of them had heterozygous mutations. Then, TA clones of the PCR products were analyzed by DNA sequencing, where a total of three types of mutations were shown (Figures 1(b) and 1(c)). We chose the third-type mutation as our research subject, as this type of mutation can cause a frameshift mutation and a stop codon will appear eight amino acids later (Figure 1(d)). Concurrently, we performed the off-target analysis using the mouse line mentioned above. The results showed no off-target effect in our experiment (data not shown). In order to produce a Myh14 homozygous mutant, F0 male mice with a 1 bp insertion were bred with wild type CBA/CaJ female, thus generating F1 mice. Then, the F1 heterozygous mice were inbred for one generation, obtaining a mouse strain homozygous for the Myh14 gene.

**Figure 2:**
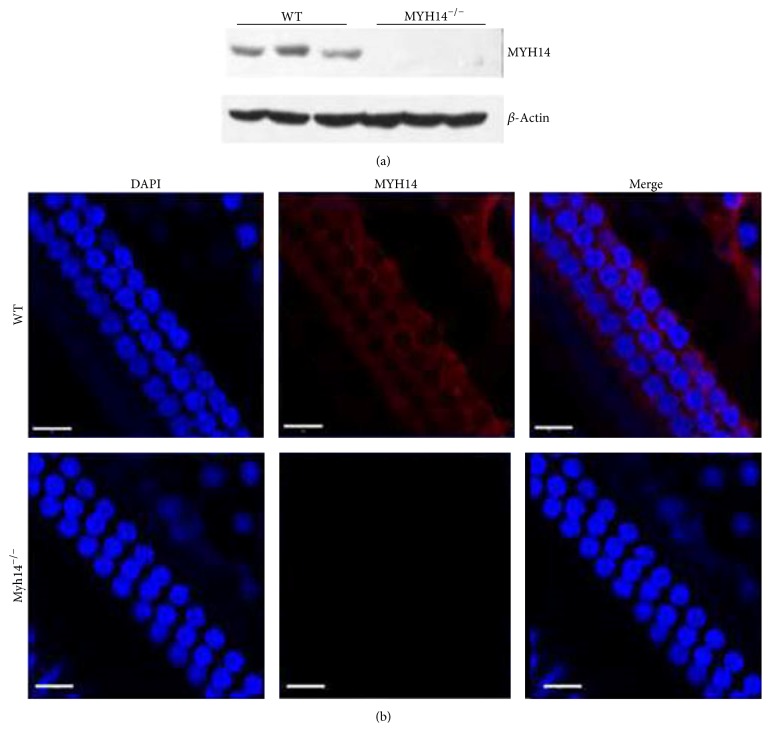
Validation of MYH14 protein knockout in mutant mice. (a) Western blot analysis validation of MYH14 protein knockout in the cerebellums of mutant mice (*n* = 3 for each group). (b) Confocal images of the basilar membrane in wild type and Myh14^−/−^ mice. The fluorescence was observed in wild type mice, but no fluorescence was seen in Myh14^−/−^ mice. Scale bar, 20 *μ*m.

To confirm that the MYH14 protein in the Myh14 mutant mice was abolished, we performed a Western blot analysis on the cerebellum (where MYH14 expression is high) using specific antibodies. As showed in Figure 2(a), MYH14 protein expression in the cerebellum was completely abolished in the homozygous mutants. Finally, we performed immunocytochemistry experiments on cochlea whole mounts (Figure 2(b)). It has been reported that MYH14 is primarily expressed in or near the reticular lamina [21]. In our study, MYH14 immunoreactivity in the apical junctional complexes (AJCs) was completely abolished in Myh14 homozygous mutants. These results consistently show the successful generation of a Myh14 knockout mouse using the CBA/CaJ strain.

### 3.2. Elevation of ABR Thresholds in Myh14^−/−^ Mice Aged Five Months

**Figure 3:**
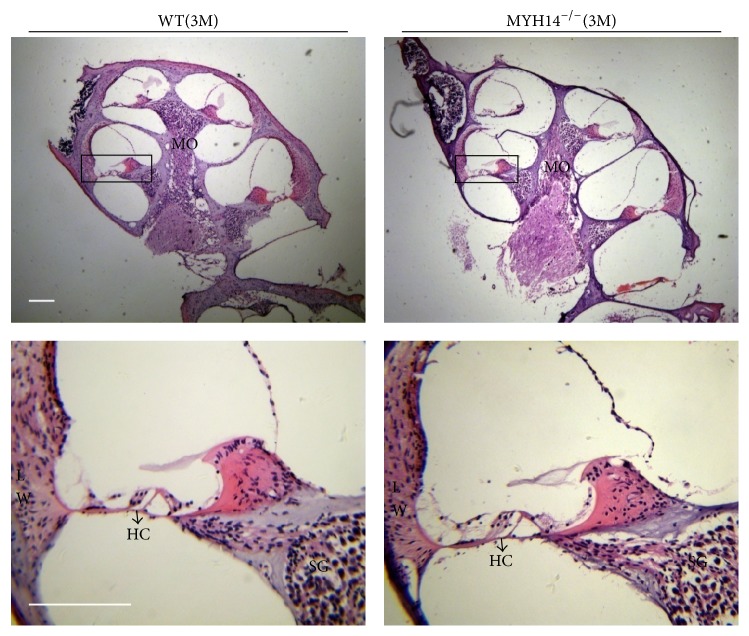
Myh14^−/−^ mice showed normal cochlear morphology. Cochlear morphology is normal in Myh14^−/−^ mutant mice. Cochlea stained with hematoxylin and eosin from 3-month-old control and Myh14^−/−^ mice. No prominent differences, including spiral ganglion (SGN) in modiolus (MO) and hair cells (HC), were found between wild type and mutant mice. Scale bar, 100 *μ*m.

**Figure 4:**
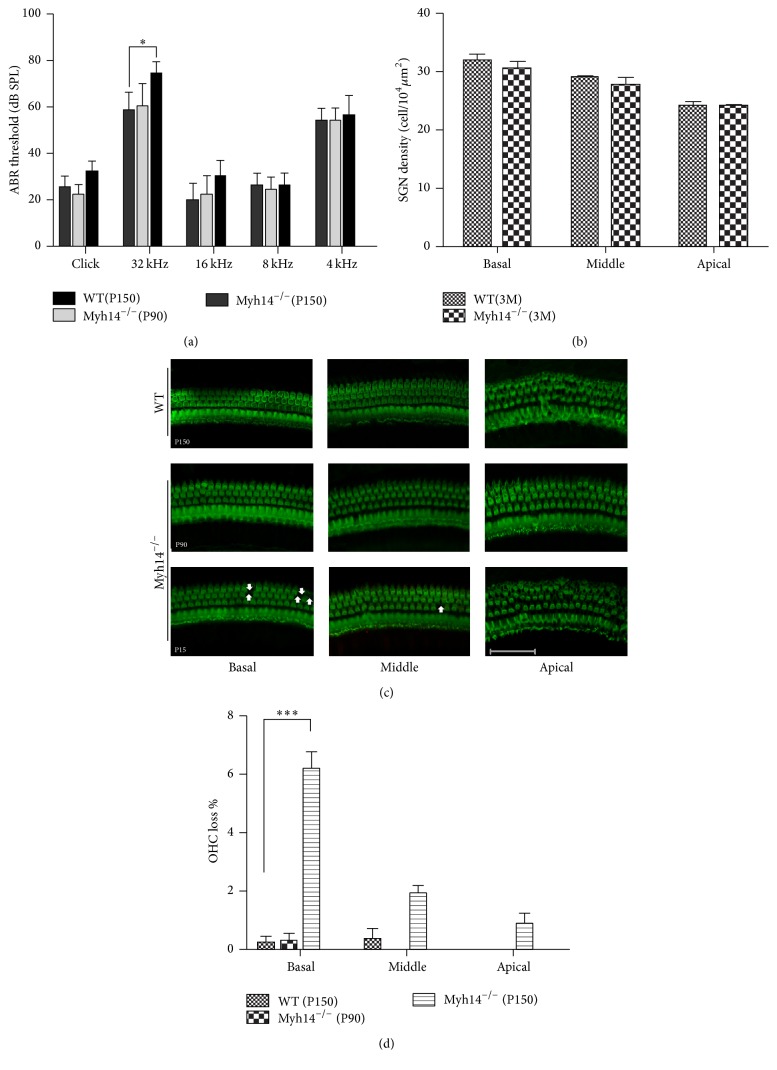
ABR measurement and cell loss patterns in cochleae of the mutant mice. (a) ABR threshold between control and Myh14^−/−^ mice (*n* = 10 for each group). ^*∗*^ *P* < 0.05 compared to the controls. (b) No significant difference in the SGN density between wild type and mutant mice. (c) Hair cell loss (white arrows) was found in 5-month-old experimental groups (*n* = 8). (d) Quantifications of OHC loss at specific locations in control and experimental groups. ^*∗∗∗*^ *P* < 0.001 compared to the control group.

Tracking analysis of Myh14^−/−^ mice was performed to determine that there were no significant differences in the appearance of Myh14^−/−^ mutant mice and wild type mice. H&E staining was performed to investigate the cochlear morphology of Myh14^−/−^ mice. The cochleae of three-month old Myh14^−/−^ mice showed no differences (Figure 3). No abnormalities were seen in either the HCs or the SGN (spiral ganglion). ABR measurements were performed in Myh14^−/−^ mice and were compared to wild type mice. Wild type mice (*n* = 10) and Myh14^−/−^ mice (*n* = 10) showed no statistical differences in ABR thresholds until five months of age (Figure 4(a)). Tone burst ABR showed that 5-month-old Myh14^−/−^ mice have high-frequency hearing loss, and the statistics show that there are significant differences. Consistent with the ABR results, immunofluorescence results showed no loss of HC and no change of SGN at three months; however, a 4%–10% HC loss was found in the cochlea basal turn of 5-month-old mutants. Sporadic OHCs were also lost in the middle and apical turn of the cochleae (Figures 4(b), 4(c), and 4(d)).

**Figure 5:**
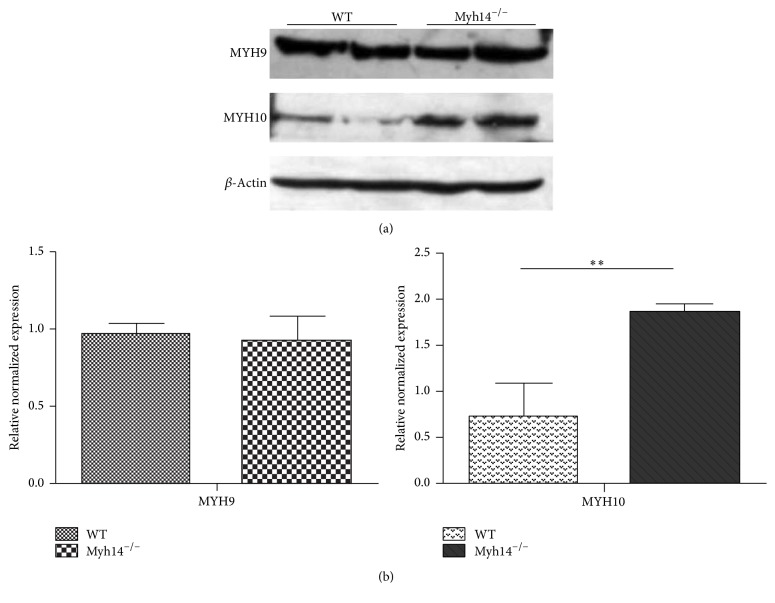
Expression of MYH10 is upregulated in the cochleae of Myh14^−/−^ mice. ((a), (b)) Western blot analysis of 3-month-old mice indicates that the expression of MYH10, not MYH9, is upregulated in the cochleae of Myh14^−/−^ mice compared to controls (*n* = 3 for each group). ^*∗∗*^ *P* < 0.01 by Student's *t*-test.

The small effect of Myh14 loss on hearing thresholds raised the possibility that other related genes might compensate for the absence of Myh14. Evidence shows that MYH14 and MYH10 are colocalized in cell-cell junctions and that MYH14 shares great similarities with both MYH9 and MYH10 in structure; therefore, they may have complementary effects in auditory function. An immunoblot analysis was performed to clarify changes in the expression of MYH10 and MYH9. Results showed that the expression of MYH10 in the cochleae of Myh14^−/−^ mice was prominent increased, but changes in the expression of MYH9 were not obvious (Figures 5(a) and 5(b)). Immunofluorescence results further verified the results of the immunoblot analysis (data not shown).

### 3.3. Expression of MYH14 Is Noise Exposure-Dependent

**Figure 6:**
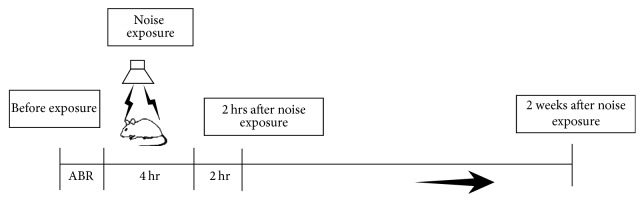
Experimental design for noise exposure and ABR test. Wild type and Myh14^−/−^ mice were exposed to noise at 105 dB for 4 h. ABR thresholds were tested at the following time points: 2 h before, 2 h after, and 7 days after noise exposure.

**Figure 7:**
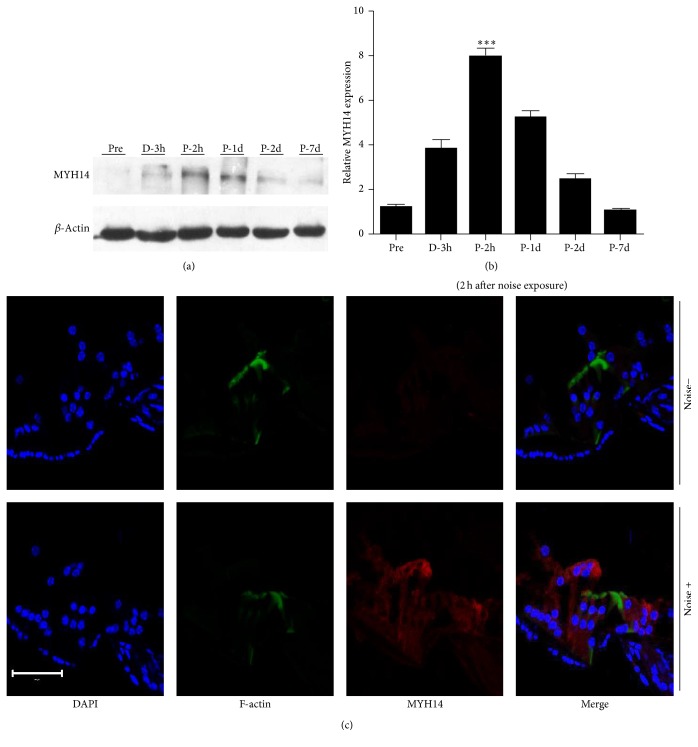
Expression of MYH14 is noise exposure-dependent. ((a) and (b)) Western blots analysis was performed at different time points (before, during, and after noise exposure) to show the expression levels of MYH14 (*n* = 3 for each group). The expression of MYH14 reached its highest peak 2 h after noise exposure (P-2h) and then gradually returned to basal levels (P-7d). ^*∗∗∗*^ *P* < 0.001 by Student's *t*-test compared to the pre-noise exposure levels. (c) Confocal images showing the expression of MYH14 before and 2 h after noise exposure in the organ of Corti. The organ of Corti was labeled with Alexa 488-conjugated phalloidin (green); red, MYH14; blue, DAPI. Scale bar, 50 *μ*m.

To investigate the response of MYH14 in mouse cochleae (2 months old) to noise exposure, Western blot analysis and immunofluorescent staining (*n* = 3 for each group) were performed to evaluate MYH14 protein levels at different time points: before (2 h), during, and after (2 and 4 h, 1, 2, and 7 days) noise exposure (Figure 6). We found that MYH14 protein expression was largely dependent on noise exposure (Figures 7(a) and 7(b)). The expression levels of MYH14 were significantly upregulated by acoustic stimuli, and they reached the highest peak 2 h after noise treatment. The results of immunofluorescence also showed that the upregulation of MYH14 was most obvious 2 h after noise treatment in the HCs of the cochlea (Figure 7(c)). However, this upregulation gradually disappeared until seven days after noise exposure, when the expression of MYH14 was restored to basal levels (before noise exposure). All these results showed that MYH14 is upregulated after noise exposure and that this change is time-dependent.

### 3.4. Myh14^−/−^ Mice Are Less Capable of Recovering from Noise Damage

**Figure 8:**
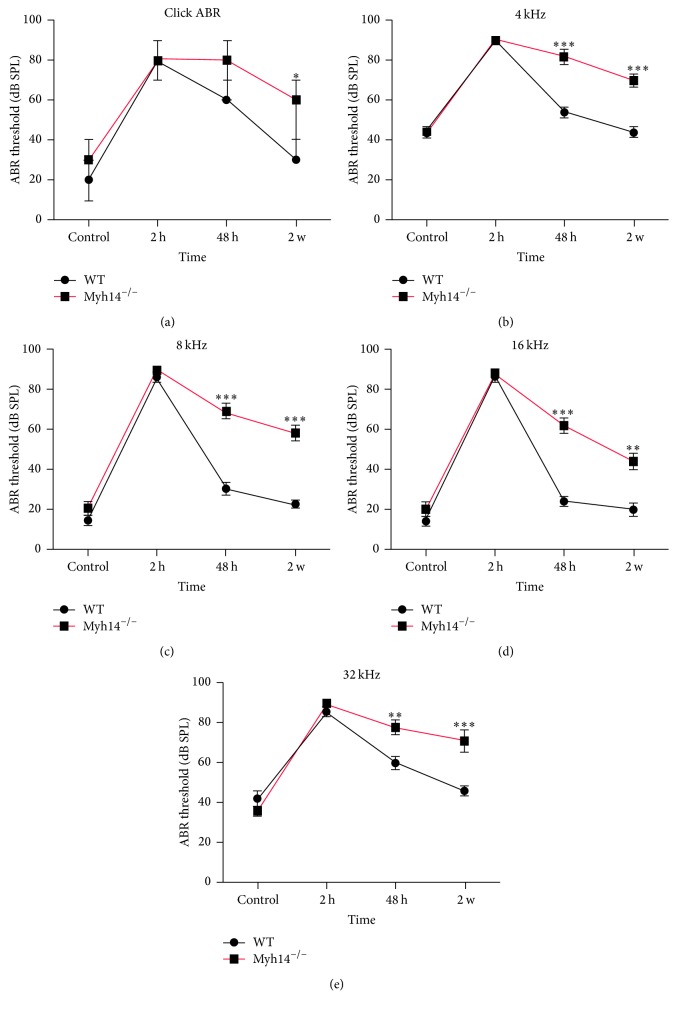
ABR threshold shifts following noise exposure between Myh14^−/−^ mice and controls. The ABR threshold shift was tested on Myh14^−/−^ mice and controls before noise exposure and 2 h, 48 h, and 2 weeks after noise exposure (*n* = 8 for each group). ^*∗*^ *P* < 0.05 compared to the controls. ^*∗∗*^ *P* < 0.01 by Student's *t*-test. ^*∗∗∗*^ *P* < 0.001 by Student's *t*-test compared to the control group. ABR measurements were reproducible.

To determine the effect of Myh14 knockout on NIHL, we examined the response to acoustic trauma in Myh14^−/−^ mice in comparison to wild type controls (4 months of age, *n* = 8 for each group). The ABR was recorded before and after acoustic trauma. The results showed that ABR thresholds were almost comparable between the two genotypes before acoustic stimuli. Then, animals were exposed to 2–10 kHz band noise at 105 db SPL for 4 h. ABR recordings 2 h after exposure showed that acoustic overstimulation induced great hearing loss (temporary threshold shift, TTS) in both Myh14^−/−^ mice and control groups; moreover, there were no significant differences between the two genotypes. The ABR test showed that wild type mice fully recovered two weeks after acoustic trauma. However, the hearing of mutant mice failed to recover two weeks after the acoustic trauma. ABR thresholds of the mutants were significantly increased compared to the controls two weeks after the high-level noise exposure (Figures 8(a), 8(b), 8(c), 8(d), and 8(e)). These results suggest that Myh14^−/−^ mice are less capable of recovering from NIHL than controls.

### 3.5. OHC Loss Was Significantly Increased 2 Weeks after Acoustic Trauma in Myh14^−/−^ Mice

**Figure 9:**
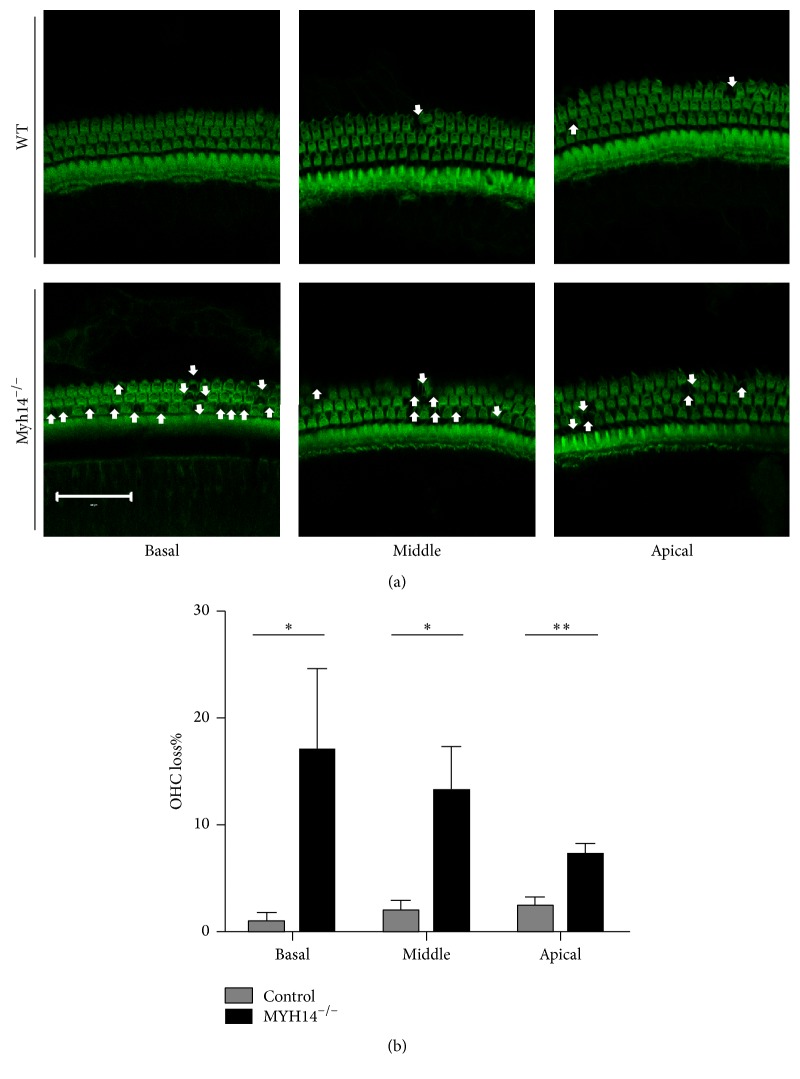
Myh14^−/−^ mice show more outer hair cell loss after noise exposure compared to controls. (a) Confocal images of outer hair cells (OHCs) in wild type and Myh14^−/−^ mice 2 weeks after noise exposure. More OHC loss (white arrow) is seen in Myh14^−/−^ mice. All of the experiments were performed at least three times. Scale bar, 20 *μ*m. (b) Percentage of OHC loss at different cochlea locations in control and Myh14^−/−^ mice. ^*∗*^ *P* < 0.05 compared to the controls. ^*∗∗*^ *P* < 0.01 compared to the controls (*n* = 5).

**Figure 10:**
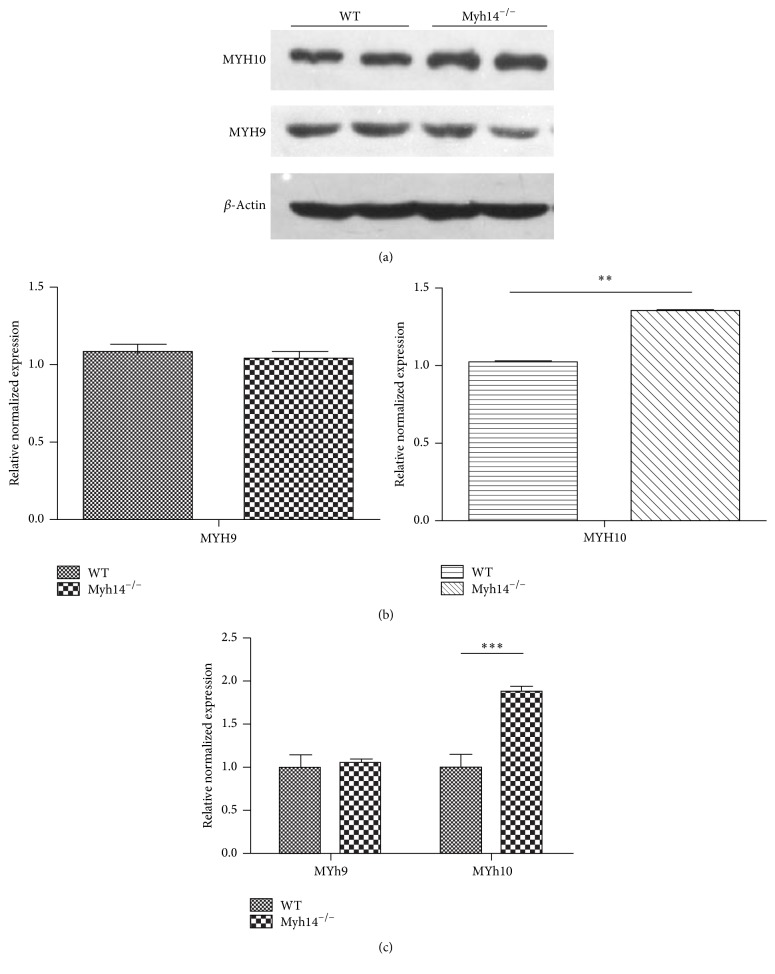
The expression level of MYH9 and MYH10 after the noise exposure. ((a), (b)) Western blots analysis was performed to show the expression level of MYH9 and MYH10 after the noise exposure (*n* = 3 for each group). (c) The consequence of Western blots was verified by real-time PCR. ^*∗∗*^ *P* < 0.01 compared to the controls. ^*∗∗∗*^ *P* < 0.001 by Student's *t*-test compared to the control group.

In order to compare the degree of damage to HCs after noise exposure in Myh14^−/−^ mice and control groups, immunofluorescence staining (phalloidin) was performed to compare HC losses of cochleae two weeks after noise exposure. The results showed that the HC loss mainly happened in the OHCs, and loss of OHCs was more significant in Myh14^−/−^ mice compared to controls (Figures 9(a) and 9(b)). However, the number of inner hair cells (IHCs) showed no obvious differences between the two groups of mice. Additionally, we did not observe defects in the spiral ganglion of either Myh14^−/−^ or control mice (data not shown). Because Myh14 is positioned at the apical junctions in mouse HCs, we examined the expression of tight-junction proteins, ZO1 and E-cadherin, in Myh14^−/−^ mice and controls after noise exposure. The immunofluorescence staining assay showed that the expressions of ZO1 and E-cadherin were not altered (data not shown). Immunoblot analysis and qRT-PCR were performed to examine if there is change in the expression level of MYH9 and MYH10. The results showed that the expression of MYH10 in the cochleae of noise-exposed Myh14^−/−^ mice was significantly increased compared with the wild type mice, while there was no obvious change in the expression of MYH9 (Figures 10(a) and 10(b)).

In summary, Myh14^−/−^ mice are less capable of recovering from noise exposure, and more HCs are lost after acoustic trauma. Our data indicate that Myh14^−/−^ mice are more vulnerable to high-level noise exposure and that Myh14 plays a protective role in noise-induced damage of OHCs.

## 4. Discussion

According to the World Health Organization (WHO) survey results, NIHL has become a very serious problem; thus, numerous efforts are being focused on understanding the mechanism of NIHL and on the treatment of hearing loss (updated February 2015). A relationship between NIHL and various genes has been established. In recent years, many NIHL susceptibility candidate genes, including MYH14, have been discovered. MYH14, which belongs to the myosin family, is required for normal hearing in humans. To this date, many mutations of the human MYH14 gene have been reported to cause DFNA4-type hearing impairment. However, the specific roles of MYH14 in auditory function and NIHL are not fully understood. In our current study, we generated a Myh14^−/−^ mice strain to clarify the role of MYH14 in the auditory system and in NIHL. Our results suggest that MYH14 plays a beneficial role after acoustic trauma.

### 4.1. CBA/CaJ Mice Are an Ideal Model for Performing NIHL and Other Hearing-Related Research

Caused by exposure to high-level noise, NIHL is one of the most common forms of sensorial hearing loss. Several experimental animal studies have reported morphological and physiological changes in the inner ear of mice after excessive noise exposure.

NIHL has a relationship with many genes. Great progress has been made in NIHL-related gene research. Currently, there are two main ways to study NIHL susceptibility genes: one is mass screening of population and the other is animal experiments. Mass screening is a commonly used method for the study of susceptibility genes in NIHL. However, population based study does not allow for the acquisition of auditory organs. In addition, to perform a study of NIHL susceptibility genes in a population, it is necessary to carry out a large-scale field survey to identify NIHL-susceptible persons. Compared with human groups, knockout mice are a good model for studying the mechanism of NIHL. In the past years, various animal knockout models have been developed. However, the establishment of traditional gene-knockout animal models must be based on traditional stem-cell methods. It is impossible to avoid using 129 and C57BL/6 background mice, which have a problem of age-related hearing loss (AHL) [20, 28]. Evidence shows that AHL and NIHL have common molecular mechanisms [29, 30], and both C57BL/6 and some 129 mice tend to be more susceptible to NIHL than CBA/CaJ mice [31]. C57BL/6 mice lose hair cells and show significant hearing loss by 6 months of age [32], and several 129 substrains except for 129Sv/Ev also show age-related hearing loss [33, 34]. Thus, this will interfere with our study of the pathogenesis of NIHL. Therefore, C57BL/6 and some 129 mice are not suitable strains for studying delayed hearing loss and NIHL. In contrast, CBA/CaJ mice retain normal hearing up to ≥18 months of age, and there is no AHL in this strain. Consequently, the CBA/CaJ mouse becomes one of the most ideal mouse strains for performing hearing-related research in the 80 inbred strains [35]. The CRISPR/Cas9 technology developed in the past three years makes the manipulation of CBA/CaJ mouse genes possible [35]. In our current study, we combined the CRISPR/Cas9 technology in the CBA/CaJ mouse strain to generate the Myh14 knockout model, thus avoiding the influence of an adverse genetic background on the experimental results.

A previous study by Ma et al. established a Myh14^−/−^ mouse using C57/B6 and 129/Sv strains. They found that there were no obvious differences between Myh14^−/−^ mice and wild type mice [23, 36]. In our current study, we generated a Myh14^−/−^ mouse using the CBA/CaJ background. In this model, no obvious differences in appearance were found; the cochleae of Myh14^−/−^ mice develop normally. However, our Myh14^−/−^ mice have high-frequency hearing loss at five months; furthermore, moderate HC loss was also found in the cochlea from the basal turn of the mutants, which also started during this time period. It is understandable that these two mouse models may have different phenotypes; a possible explanation is that the mouse strain used in this study is not the same as the one used in the previous study, and the susceptibility to NIHL is different between strains [37, 38].

### 4.2. MYH10 May Compensate for the Absence of MYH14 in the Cochleae

Myosin is a superfamily of proteins related to the movement of molecules that are present in all eukaryotic cells; it binds to actin and uses ATP to move along the actin filament. The myosin superfamily is divided into two major categories: traditional myosin (nonmuscle myosin II; NM II) and nonconventional myosin (myosins I and III–VII). The distribution of the myosin families in the cochlea is different, and there is a difference in their normal physiological functions in the cochlea. NM II is thought to be involved in mediating epithelial tissue morphogenesis and tensional homeostasis, regulating force within epithelial apical junctions [39–41]. There are three isoforms of NM II in the cochlea, encoded by* myh9*,* myh10*, and* myh14* in mice [21]. The three NM II isoforms share very similar molecular structures [22]. Therefore, the slight effect of MYH14 loss on hearing thresholds raised the possibility that the other two genes may compensate for its absence [36]. The immunoblot analysis result showed that only the expression of MYH10 was significantly increased, and there was no obvious change in the expression of MYH9. A previous study reported that MYH10 is the major isoform regulating Schaffer collateral inputs [42]; thus, it is likely that the increase in MYH10, but not MYH9, can compensate for most of the functions of MYH14 in cochlea. The results from the MYH14 gene screening revealed one nonsense mutation. The affected individuals exhibited progressive hearing impairment beginning in the 1st decade of life with profound hearing loss in the 4th decade. This is to contrast with our Myh14^−/−^ mice where onset of progressive hearing loss began at 5 months of age. This milder phenotype observed with our Myh14^−/−^ mice could be explained by a more pronounced compensatory effect of MYH10 on MYH14 in the murine model. However, MYH10 cannot completely replace the function of MYH14 because loss of HCs in Myh14^−/−^ mice was still observed from five months. Moreover, we cannot exclude the possibility that other unidentified molecules may compensate for MYH14.

### 4.3. Loss of MYH14 Promotes NIHL

Evidence from several mouse mutants showed that the pathogenesis of AHL and NIHL is highly similar in most cases [17, 18]. In our study, Myh14^−/−^ mice were used to investigate the role of MYH14 in the mechanism of NIHL. We found that abrogation of Myh14 increased the mouse susceptibility to acoustic trauma. In the wild type mice, 105 dB SPL noise exposure for 4 h caused a TTS, and their ABR thresholds recovered two weeks after noise exposure. However, mutant mice exhibited impaired recovery of ABR thresholds. In addition, more HC loss (mainly OHCs) was observed in mutant mice compared to controls two weeks after noise exposure. Consistent with previous reports [43–45], our results indicated that OHCs are more vulnerable to noise compared to IHCs. Failed recovery of ABR thresholds in mutant mice may be partially caused by permanent HC loss in the cochlea.

In addition, we found no obvious differences in the spiral ganglion and lateral wall (two other places easily affected by noise exposure) between Myh14^−/−^ and control mice after noise exposure. Thus, our results indicated that MYH14 plays an indispensable role in HCs, when compared to that in other parts of the cochlea. Together, these findings suggest that MYH14 may play a beneficial role in the protection of the cochlea after acoustic stimulation in the CBA/CaJ mouse line.

We tried to determine the mechanism through which Myh14^−/−^ mice were susceptible to NIHL. Because MYH14 is positioned at the apical junctions in the mouse HCs, we studied the expression of ZO-1 and E-cadherin cell junction proteins in Myh14^−/−^ mice after noise exposure using immunofluorescence analysis to then compare them to the control group. We found that there were no statistically significant differences between both of these proteins in the Myh14^−/−^ mice when compared to the controls. Then, how does a MYH14 deficiency cause more HC loss after noise exposure? A possible explanation is that loss of MYH14 can damage the permeability of ions in a noisy environment. Under normal conditions, MYH10 can partially compensate for the function of MYH14, but the absence of MYH14 makes it easier for mutant mice to be damaged in AJCs compared to the controls in a noisy environment. AJCs with long-term damage may cause changes in ion concentration, which eventually induced more HC loss in Myh14^−/−^ mice [46, 47]. However, the specific mechanism of this hypothesis needs to be further analyzed.

## 5. Conclusion

We used the CRISPR/Cas9 technology in the CBA/CaJ mouse strain to generate a MYH14 knockout model. Myh14^−/−^ mice were more vulnerable to high intensity noise compared to control mice. MYH14 may play beneficial role in NIHL. Additional experiments are necessary to identify the mechanism through which MYH14 contributes to NIHL.
